# A reminder of the association between *Clostridium septicum *and colonic adenocarcinoma

**DOI:** 10.1186/1477-7800-3-12

**Published:** 2006-04-28

**Authors:** Azhar A Khan, Kim Davenport

**Affiliations:** 1 Department of surgery, Stafford General Hospital, Weston Road, Stafford, ST16 3SA, UK

## Abstract

We present the case of a patient, with previously unknown liver metastases, presenting with a liver abscess and *Clostridium septicum *septicaemia. *C. septicum *is known to be associated with both malignancy and immunosuppression and therefore in patients where this organism is isolated, efforts must be made to exclude an occult underlying malignancy or haematological disorder.

## Introduction

Severe infection with *Clostridium septicum *in healthy humans is relatively rare. The organism used to be a well known complication of war wounds in the form of gas gangrene [1]. *C. septicum *can cause other rigorous focal or disseminated infections by spontaneous invasion from the gut of compromised patients. *C. septicum *produces exotoxin which is responsible for rapid progression of infection. Exotoxin hydrolyses cell membranes, causes tissue necrosis by inducing occlusive microvascular thrombosis. These spontaneous forms of infection are believed to be associated with colonic malignancy (especially in the cecum) [2], acute leukemia or cyclical neutropenia [3]. Unlike *C perfringens, C septicum *is aerotolerant and can infect normal tissues. We are reporting this recent case as a reminder of this association and to re-emphasise the importance of investigating these patients for occult malignancy.

## Case report

**Figure 1:**
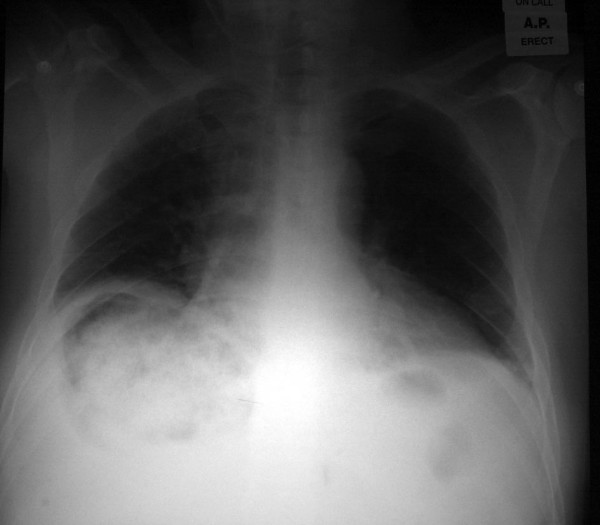


A 59 year old gentleman presented with a 3 day history of right upper quadrant pain radiating into the right shoulder. His only past medical history was hypertension. On examination, he was jaundiced with a temperature of 38.2°C, pulse 120 and a blood pressure of 130/80. He was found to have tender hepatomegaly. His blood results showed an anaemia of 9.6, mild renal impairment (urea 11.9, creatinine 142) and hepatic impairment (Bilirubin 60, AST 274, alkaline phosphatase 170). An X-Ray [figure 1] revealed multiple gas filled lesions within the right upper quadrant of the abdomen which were confirmed on ultrasound as originating in the liver.

**Figure 2:**
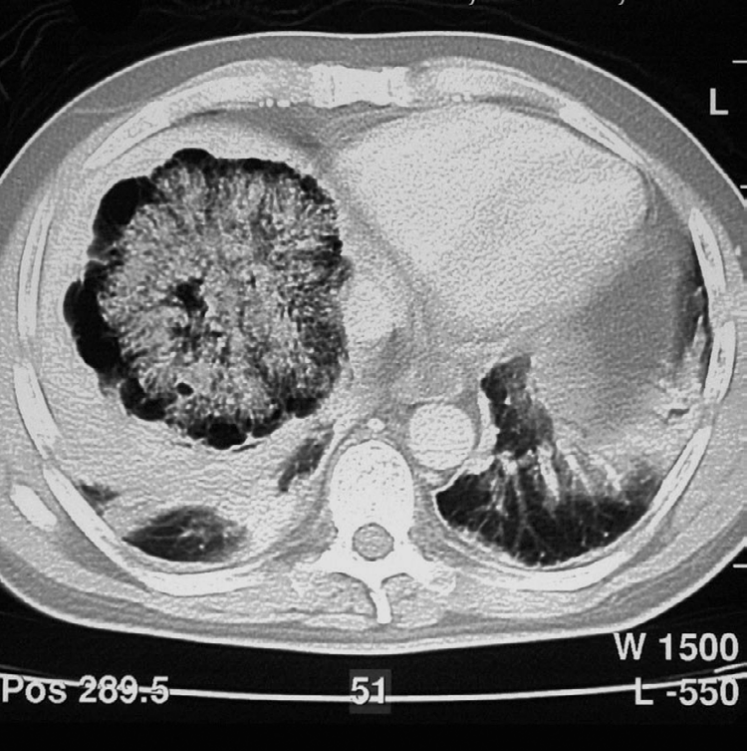


CT [figure 2] revealed the presence of a central core of irregular soft tissue in the liver, which was thought to be an abscess. A drain was inserted under ultrasound guidance. Both the blood cultures and drainage fluid grew *Clostridium septicum*. He proceeded to have an ultrasound-guided biopsy of the central soft tissue core. Histology confirmed a necrotic metastatic adenocarcinoma with morphology consistent with a colorectal origin. Immunohistochemistry further supported this assumption as cytokeratin 7 was negative and both cytokeratin 20 and CEA were positive. Serum tumour markers revealed an elevated CEA (14) and CA19-9 (66).

## Discussion

*Clostridium septicum *is an anaerobic, gas forming, gram-positive bacillus. It is thought to be an intestinal commensal in man and animals. It is believed that the caecum and terminal ileum provide the most favourable environment for colonisation. The presence of mucosal barrier disruption, for example, mucosal ulceration secondary to malignancy, ischaemia or chemoradiotherapy, is thought to allow invasion into the blood stream. The gastrointestinal tract is the presumed portal of entry in the majority of cases.

Infections with this organism are rare but are associated with serious outcomes with a mortality of up to 45–70 %. It produces marked toxicity and may present as gas gangrene, myonecrosis, sepsis or liver abscesses. *C. septicum *is rarely able to cause liver abscesses in the absence of any underlying pathology [4]. Most typically the underlying lesion is a metastasis, which has outgrown its blood supply and therefore provides an ideal anaerobic environment for bacterial growth. The gas produced by the organism appears to remain limited to the area of metastases and does not invade adjacent healthy tissue [4] so causing generalised liver enlargement. To the best of our knowledge, this organism has not been reported with primary liver carcinoma in the literature.

There is a known association between *C. septicum *and immunosuppression or malignancy, most commonly of colonic or haematological origin, although there are reported cases in association with choriocarcinoma [5] and breast carcinoma [6] in the literature. In one study [7] looking at all *Clostridial *infections, 11% were secondary to C. *septicum*, an associated malignancy was found in 50% (as compared to 11% of patients with other clostridial infections) and the remaining patients all had evidence of immunosuppression. There was 56% mortality with C. septicum as opposed to 26% with other strains. In another [8], 87.5% of cases were found to have malignancy, 86% of these originated in the gastrointestinal tract and the remainder were haematological.

Intravenous antibiotics, predominantly penicillin, remain the mainstay of treatment, and in some cases, surgical debridement is necessary.

## Conclusion

Those patients found to have *C. septicum *should undergo an aggressive search for an underlying malignancy or haematological abnormality if they are fit for further interventions.

## Conflict of interest

The author(s) declare that they have no competing interests.
